# Respiratory Epithelial Adenomatoid Hamartoma: An Important Differential of Sinonasal Masses

**DOI:** 10.7759/cureus.2495

**Published:** 2018-04-17

**Authors:** Darren Rom, Migie Lee, Edward Chandraratnam, Ronald Chin, Niranjan Sritharan

**Affiliations:** 1 ENT Department, St. George Hospital, University of Sydney, Sydney, AUS; 2 ENT Department, Nepean Hospital, Kingswood, AUS; 3 Department of Pathology, Westmead Hospital

**Keywords:** hamartoma, sinonasal, histopathology, mass, differential

## Abstract

Background

Respiratory epithelial adenomatoid hamartomas (REAH) are rare, glandular proliferations of the aerodigestive tract lined by ciliated respiratory epithelium. We report nine cases of REAH and devised a histopathological guide to differentiate these lesions from its main differentials.

Methods

Patients with biopsy-proven REAH were included in the series. Lesions were removed endoscopically and sent for histopathological analysis. The macroscopic and microscopic features were reviewed.

Results

Nine patients (age 59 ± 15.5 years, 78% male) with REAH were analysed. Findings revealed glandular proliferations lined by ciliated respiratory epithelium without metaplastic changes and intervening oedematous stroma. This is in contrast to a typically thickened epithelial basement membrane with oedematous stroma seen in nasal polyps.

Conclusion

REAHs are benign entities that should be included in the differential diagnosis for sinonasal masses. Prompt detection by tissue biopsy is crucial to differentiate these lesions from nasal polyps and more aggressive pathologies and avoid unnecessary surgery.

## Introduction

Respiratory epithelial adenomatoid hamartomas (REAHs) are benign, glandular proliferations of the surface epithelium of the nasal cavity and paranasal sinuses, which were first reported by Wenig and Heffner in 1995 [1]. While these lesions have been well described in a number of cases since that original report, there is still much debate surrounding its aetiology and pathogenesis. Two separate entities have been reported, those associated with nasal polyps and solitary lesions, the latter being more uncommon. REAH occurring in the setting of nasal polyps suggests sinonasal inflammation may be an important pathophysiological factor [2, 3]. It is becoming increasingly necessary to differentiate REAH from other sinonasal masses such as nasal polyps or more sinister pathologies like inverted papillomas, all of which can mimic these lesions clinically and radiologically [4]. Histological characterisation remains the gold standard for diagnosis, the clinical significance of which is paramount to avoid unnecessary and aggressive surgery for an otherwise benign lesion. The objective of this study was to report the histopathological characteristics of patients with REAH and compare these with the microscopic findings of patients with nasal polyps. A table summary showing this comparison is described. We also report the clinicopathological findings of REAH and compare this against the current literature.

## Materials and methods

A retrospective case series of patients diagnosed with REAH at Austpath Laboratories was performed from 2014 to 2017. Patient demographics, clinical presentation and intraoperative findings were reported. In all cases, the mass was removed by transnasal endoscopic resection. Specimens were collected at the time of surgery, fixed in formalin and sent for histopathological analysis. The entire specimen was processed in all cases except one, in which representative sections were sampled. After routine dehydration and embedding, all tissue blocks were sectioned at 4 μm and stained with haematoxylin and eosin (H&E). The macroscopic and microscopic features were reported.

## Results

Patient demographics

Nine patients with REAH were reported. 78% were male (n = 7) and mean age was 59.4 ± 15.52 years. The presence of concurrent nasal polyps was evident in 67% (6/9) and as a solitary lesion in 33% (3/9). The nasal cavity was implicated in 89% (8/9) of cases, while the olfactory cleft was reported in one case. Notably, olfactory involvement occurred in the context of a solitary sinonasal mass. The macroscopic appearance was described as polypoid in seven cases and irregular in two. The size of the specimen ranged from 4 mm to 45 mm (average of 14.1 mm).

Histopathological characteristics

**Figure 1:**
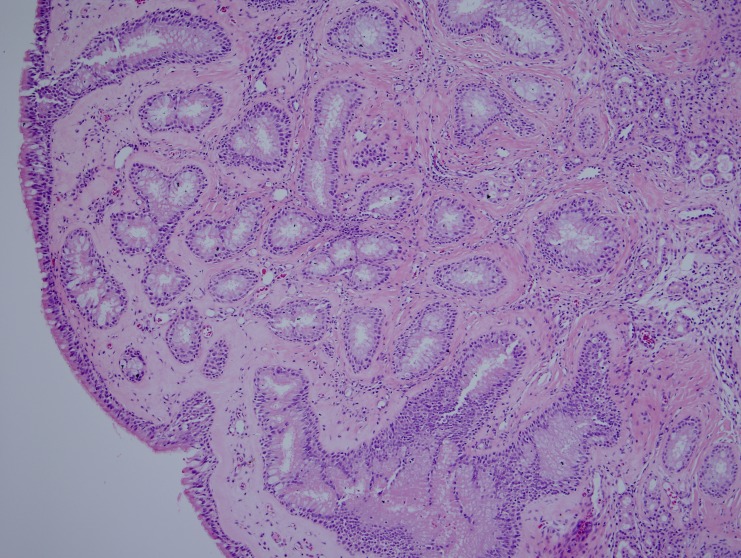
Histological characteristics of REAH. A low powered photomicrograph showing small- to medium-sized glands separated by stroma. REAH: Respiratory epithelial adenomatoid hamartoma

**Figure 2:**
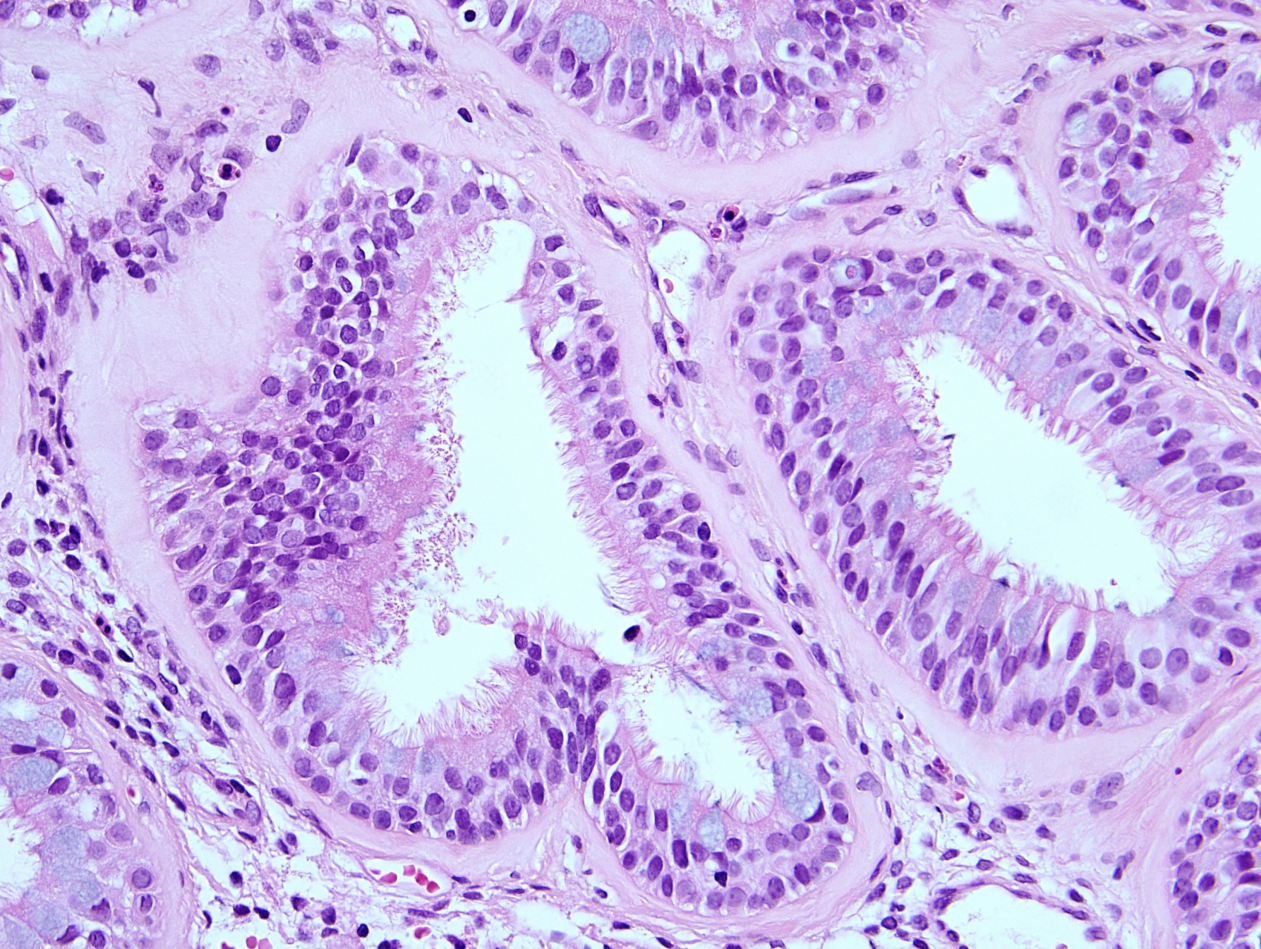
High powered photomicrograph showing multi-layered ciliated epithelium with hyaline thickening of the basement membrane.

In most of the cases (seven of nine), the polypoid mucosal fragments showed prominent glandular proliferation. These glands were widely separated, small to medium sized, round to oval shaped and separated by stroma (Figure 1). In two cases, variably dilated glands were present and cystically dilated glands filled with mucin were occasionally noticed in one of these two cases. The glands were lined by multi-layered ciliated respiratory epithelium in which variable numbers of goblet cells were identified (Figure 2). Prominent hyaline thickening of the basement membrane of the glands was a typical finding. Focal immature squamous metaplasia was noticed in one case. The stroma was mild to moderately oedematous and infiltrated by small to large numbers of lymphocytes and plasma cells. Eosinophils in small numbers were present in a few samples. In two cases the features described above occurred in the core of an otherwise typical inflammatory sinonasal polyp. In one case, there was a lack of the characteristic hyaline thickening of the basement membrane but all other features were present.

## Discussion

REAH remains an important differential in the context of sinonasal masses and can often be difficult to distinguish on clinical or radiological grounds alone. Furthermore, what was once thought to be an exceptionally rare entity is now considered to be more prevalent, especially in the context of nasal polyps. This may be due to an increased awareness of the condition by clinicians as more cases are being reported in the literature, but also a greater number of incidental findings on histopathological diagnosis.

REAH, whether found in isolation or in association with nasal polyps, demonstrates typical histopathological characteristics [5]. Glandular proliferation distended with mucous and separated by stroma is a dominant feature, as well as the surface epithelium that invaginates into the submucosa and in direct continuity with the glands. A thickened, hyalinised glandular basement membrane that envelopes the proliferating glands is another characteristic finding unique to REAH. In contrast, nasal polyps do not demonstrate glandular proliferation nor surface epithelial invagination, and more typically show a thickened epithelial basement membrane whereas in REAH this is absent. While REAH usually affects the posterior nasal septum, inflammatory polyps rarely show septal involvement. The key histopathological features of REAH and nasal polyps are summarised in Table 1.

**Table 1:** Comparison of histological characteristics of REAH, nasal polyps and inverted papillomas. HPV: Human papillomavirus; REAH: Respiratory epithelial adenomatoid hamartoma.

Histological features	REAH	Nasal polyp	Inverted papilloma
Site	The majority occurs in the nasal cavity. Usually affects the nasal septum, especially in the posterior area. Usually unilateral.	Rarely show septal involvement. Usually bilateral.	Most common in nasal cavity and maxillary sinus. About a third of cases originate from multiple sites. Rarely bilateral.
Surface epithelium	Invaginates into submucosa and direct continuity with proliferating glands can be seen.	Surface invagination absent and characteristically basement membrane is thickened.	Multiple inversions of the surface epithelium into the underlying stroma.
Basement membrane thickening	Absent	Present	Absent
Glandular proliferation	Widely spaced, small- to medium-sized, round to oval glands separated by stroma (dominant feature). Glands are usually distended with mucus.	Glandular proliferation absent.	Multiple inversions of the surface epithelium into the underlying stroma with continuous, distinct and intact basement membrane.
Lining epithelium of glands	Respiratory epithelium often with admixed mucin-secreting (goblet) cells. Atrophic glands lined by single layer of flattened to cuboidal-epithelium may be present.	Glandular proliferation absent.	Proliferating squamous and/or respiratory cells with numerous microcysts (infiltration of epithelium by transmigrating neutrophils). Non-keratinizing squamous or transitional epithelium (5-30 cells thick), frequently predominates and is covered with a layer of ciliated columnar cells. Occasionally spare mitoses confined to the basal layers.
Basement membrane of glands	Hyalinization of variably thickened, eosinophilic basement membrane enveloping proliferating glands (characteristic finding).	Thickening of basement membrane of glands absent	Distinct and intact, continuous basement membrane.
Stroma	Oedematous or fibrous stroma containing mixed inflammatory cell infiltrate (plasma cells, lymphocytes)	Marked stromal oedema and mixed inflammatory cell infiltrate (eosinophils, plasma cells and lymphocytes), bland-appearing stromal fibroblasts and small- to medium-sized blood vessels	Either loose or dense, and maybe inflamed.
Possible additional findings	Co-existence with inflammatory polyps can occur. Tissues native to sinonasal tract or nasopharynx such as adipose tissue, bone, cartilage and chondromesenchymal tissue can occur rarely. Co-existence with Schneiderian papilloma (inverted type) and solitary fibrous tumour can occur rarely.	Atypical stromal cells, granulation tissue, granuloma formation, amyloid-like stroma can occur rarely.	Premalignant and malignant features: dysplasia, carcinoma in situ, invasive carcinoma can occur rarely. Thorough sampling and evidence seeking for malignant transformation should always be performed. HPV infection can be detected in a number of cases.

Other clinically important differential diagnoses for REAH include Schneiderian papillomas of the inverted type and low-grade adenocarcinomas. Macroscopically, these more aggressive lesions can resemble the benign hamartoma especially in its solitary form, and so histological characterisation is almost always required. Inverted papillomas exhibit invagination of hyperplastic squamous and/or respiratory epithelium surrounded by a thin basement membrane. The inverted growth of squamous epithelium is not found in REAH, yet this can be difficult to identify to the inexperienced pathologist.

REAH occurs in adults with a strong male to female preponderance and typically affects those in their fifth and sixth decade of life. We report an average age of 59 years and males being affected in 80% of cases. This is in keeping with the original report of 31 patients by Wenig and Heffner who described a mean age of 58 years with 87% patients being male, as well as a larger study by Vira et al. who reported a mean age of 52 years with 57% being male [1, 6]. The majority of cases in this study found REAH to be associated with nasal polyps (67%) and this again is similar to other larger case series. Hawley et al. demonstrated a 79% polyp association in a series of 45 patients [2].

REAHs can present with similar signs and symptoms of the chronic inflammatory disease, which usually occur gradually and with a long duration, lasting from months to years. These include nasal obstruction, rhinorrhoea, facial pressure, headaches, and altered sense of smell [6]. Endoscopically, they can appear as pink, fleshy, distinct masses, but with no characteristic features that can distinguish it from other sinonasal lesions [7].

There is no clear etiologic factor in the development of REAH, however, the frequent association with nasal polyps suggests an inflammatory component [8]. Lorentz et al. demonstrated the presence of REAH in 48% of biopsied oedematous olfactory clefts associated with nasal polyps and it has thus been theorised that a state of chronic inflammation in the nasal cavity may lead to the development of this lesion [9].

Despite this, reports of solitary REAHs do not exhibit a co-existing inflammatory process, and others theorise that perhaps inflammation occurs secondary to the growing mass [10, 11]. Furthermore, REAH has typically been described to occur in the posterior septum, a site uncommon for nasal polyps [1, 6]. Other hypotheses to explain its development include a congenital malformation as its name suggests, or that it exists along the spectrum of a benign neoplasm [12]. In one study that addressed the molecular changes of REAH, there was an unusually similar profile to that of sinonasal adenocarcinomas, including a loss of heterozygosity found on chromosomes 9p and 18q as well as fractional allelic loss of 31% [13].

Treatment of REAH is usually curative with endoscopic resection and recurrence following is extremely rare [2]. Despite its slow growth and lack of malignant potential, surgery is still indicated firstly for a formal diagnosis but also the possibility of orbital extension and intracranial involvement [14]. The recurrence rates of REAH are rare, with one report in the literature being 3.7% after an average of 3.8 years [6]. No studies have investigated the long-term recurrence rates. Histological misdiagnosis with inflammatory polyps, inverted papillomas or low-grade sinonasal adenocarcinomas may lead to more extensive and unnecessary intervention, while mistaking these differentials for REAH may lead to incomplete resected margins [6].

## Conclusions

REAH is a benign entity with hallmark histological features of glandular proliferation, surface epithelium that invaginates into the submucosa and oedematous intervening stroma. It is often found incidentally and given its low prevalence, can often be mistaken for other sinonasal masses seen clinically including inflammatory nasal polyps and true neoplasms like inverted papillomas and low-grade sinonasal adenocarcinomas. Surgeons predominantly rely on formal histological characterisation in order to avoid unnecessary or aggressive treatment.
